# The Forthcoming Harvest of Doctors—Some Suggestions

**Published:** 1881-02

**Authors:** 


### Editorial.

### THE FORTHCOMING HARVEST OF DOCTORS—SOME SUGGESTIONS.

During the present month the lecture term of many of the medical colleges will close, and there promises to be an abundant harvest of candidates for the honors of the medical degree. We are not, however, as much concerned with the *quantity*, for the law of supply and demand for many years has failed of application in this matter, as we are deeply interested in the *quality* of those who are to be entrusted with the responsibility imposed by the medical profession upon its members. Into the solution of this very important problem, which influences so much the present and prospective interests of the profession, there enter two factors—first, the practitioner under whom the student passes his pupilage, and, second, the medical college in which authority is vested to confer the degree of Doctor of Medicine. Whatever may be said of the laxity of the medical schools in the matter of medical education, they are, taken as a whole, but the reflex of the prevailing sentiment of the profession on this vital question. The college, as a general rule, opens its doors and adapts its curriculum and its standard to the circumstances and conditions, imposed by the profession, which fosters and maintains it. Without endowment, it is not inclined to encourage its faculty in lecturing to empty benches; hence, on business principles, its standard is arranged for such students as the profession supply.

Now we say that the profession at large have a grave responsibility in the matter of medical education, which they must share with the medical schools, that simply take such material as is offered them. Further, it is evident that inasmuch as the schools are or ought to be controlled by the best talent of the locality in which they exist, men in advance in professional attainments and reputation of their fellows, it is incumbent on them to be also in advance, indeed to lead the vanguard in the work of raising the standard of medical education. The question is often presented to reflecting minds, Do the colleges meet this great responsibility? We are forced to acknowledge that they often fail to come up to the full measure of their duty. As to the character of the instruction they impart in the various departments of medical science, we have little to say. A late writer remarks that the schools furnish "too little knowledge and too much information." This criticism is a just one, if we regard knowledge a familiarity with things, and information no more than a memory of words. The information imparted in many of the lectures fails of great practical utility, on account of the absence of opportunities to the student for work at the bedside. Besides the knowledge or information is imparted in many instances to minds uncultured and inadequately trained to receive and comprehend the principles the teacher aims to impress.

The remedy for this state of things rests, first with the profession, which must be more select in their choice of students, and then with the schools, which should lead the profession step by step to an elevated standard, and to a higher appreciation of medicine as a science, seeking the best intellectual culture and attainments for its members.

In what we have said we intend more than a general, rather a local application, in which we would not exclude the excellent medical school of this city. If we acknowledge that we have a just pride in its reputation, we are only guilty of the candor, due as journalists, to an institution which has furnished many honorable and conspicuous names to the American profession. If we also confess that its standard ought, in justice to itself and the profession, to be raised in the examinations for the medical degree, which are soon to take place, we are only stating that which its faculty, composed of men of acknowledged ability and sagacity, can afford to grant. The public and the profession may well expect from such a source an elevated standard, and they in return are in a position to more than meet the requirement.

A step further, and we hope not long to await its fulfillment, is the introduction of the graded course of medical study, such as Harvard has adopted. The profession should encourage the schools in establishing this system. A lengthened term of study, with daily recitations and frequent examinations and an elevated standard of requirements for the medical degree, are conditions which the profession should accept, and the schools adopt. We would not expect too much, nor such a revolution in the study of medicine too early, but wish to hold up to our own school, as well as to other colleges, the high aim to which earnest efforts should be directed.

